# Tumor-Associated Circulating MicroRNAs as Biomarkers of Cancer

**DOI:** 10.3390/molecules19021912

**Published:** 2014-02-10

**Authors:** Jin Wang, Ke-Yong Zhang, Song-Mei Liu, Subrata Sen

**Affiliations:** 1 Department of Translational Molecular Pathology, The University of Texas MD Anderson Cancer Center, Houston, TX 77030, USA; 2 Department of orthopedics, Daye People’s Hospital, Daye, Hubei 435100, China; 3 Center for Gene Diagnosis, Zhongnan Hospital of Wuhan University, Wuhan, Hubei 430071, China

**Keywords:** miRNA, circulating miRNA, body fluid, biomarker, cancer

## Abstract

MicroRNAs (miRNAs), the 17- to 25-nucleotide long noncoding RNAs that modulate the expression of mRNAs and proteins, have emerged as critical players in cancer initiation and progression processes. Deregulation of tissue miRNA expression levels associated with specific genetic alterations has been demonstrated in cancer, where miRNAs function either as oncogenes or as tumor-suppressor genes and are shed from cancer cells into circulation. The present review summarizes and evaluates recent advances in our understanding of the characteristics of tumor tissue miRNAs, circulating miRNAs, and the stability of miRNAs in tissues and their varying expression profiles in circulating tumor cells, and body fluids including blood plasma. These advances in knowledge have led to intense efforts towards discovery and validation of differentially expressing tumor-associated miRNAs as biomarkers and therapeutic targets of cancer. The development of tumor-specific miRNA signatures as cancer biomarkers detectable in malignant cells and body fluids should help with early detection and more effective therapeutic intervention for individual patients.

## 1. Introduction

One of the major challenges in cancer research is the identification of stable biomarkers that can be routinely measured in easily accessible samples. Serum tumor markers, such as carcinoembryonic antigen and carbohydrate antigen 19-9, are being used in convenient diagnostic assays [1,2]. Among the growth factors involved in cancer progression, several angiogenic factors, such as vascular endothelial growth factor and basic fibroblast growth factor, have drawn attention as candidate biomarkers for detection of cancer [3]. However, these conventional serum markers lack sufficient sensitivity and specificity to facilitate early detection of cancer. During the past decade significant attention has been paid to cell-free nucleic acids (cfNAs), such as DNA, mRNA and miRNA, which are present at varying concentrations in the blood of cancer patients. cfNA yields are higher in patients with malignant lesions than in patients without tumors, but increased levels have also been quantified in patients with benign lesions, inflammatory disease and tissue trauma. The physiological events that lead to the increase of cfNA during cancer development and progression are not well understood. However, analyses of circulating DNA have allowed the detection of tumor-related genetic and epigenetic alterations that are relevant to cancer development and progression. The release of nucleic acids into the blood is thought to be related to the apoptosis and necrosis of cancer cells in the tumor microenvironment. Secretion has also been suggested as a potential source of cell-free DNA (cfDNA) [4]. The circulating nucleic acids are present in serum and other body fluids and may represent potential biomarkers. It has been almost 60 years since the first study appeared demonstrating differences in levels of cfDNA between healthy and sick individuals [5]. The increased levels of blood cfDNA in a number of diseases indicate that cfDNA can be used as a noninvasive, rapid, sensitive and accurate method of diagnosis of human diseases, including cancer. An elevated level of cfDNA has been detected in breast [6], colorectal [7], liver [8,9], lung [10], ovarian [11], prostate [12], esophageal [13], gastric [14], rectal [15] and endometrial cancer [16].

Tumor-specific miRNAs were first discovered in the serum of patients with diffuse large B-cell lymphoma (DLBCL); high levels of *miR-21* correlated with improved relapse-free survival [17]. We found that more than 150 studies have assessed the potential use of serum or plasma miRNAs as biomarkers in different types of cancer. Although unique expression profile of serum miRNAs has been identified in different cancer types, similar profiles of circulating miRNAs have also been implicated to reflect physiological roles in development and disease [18].

## 2. Biology, Biogenesis and Function of miRNAs

**Figure 1:**
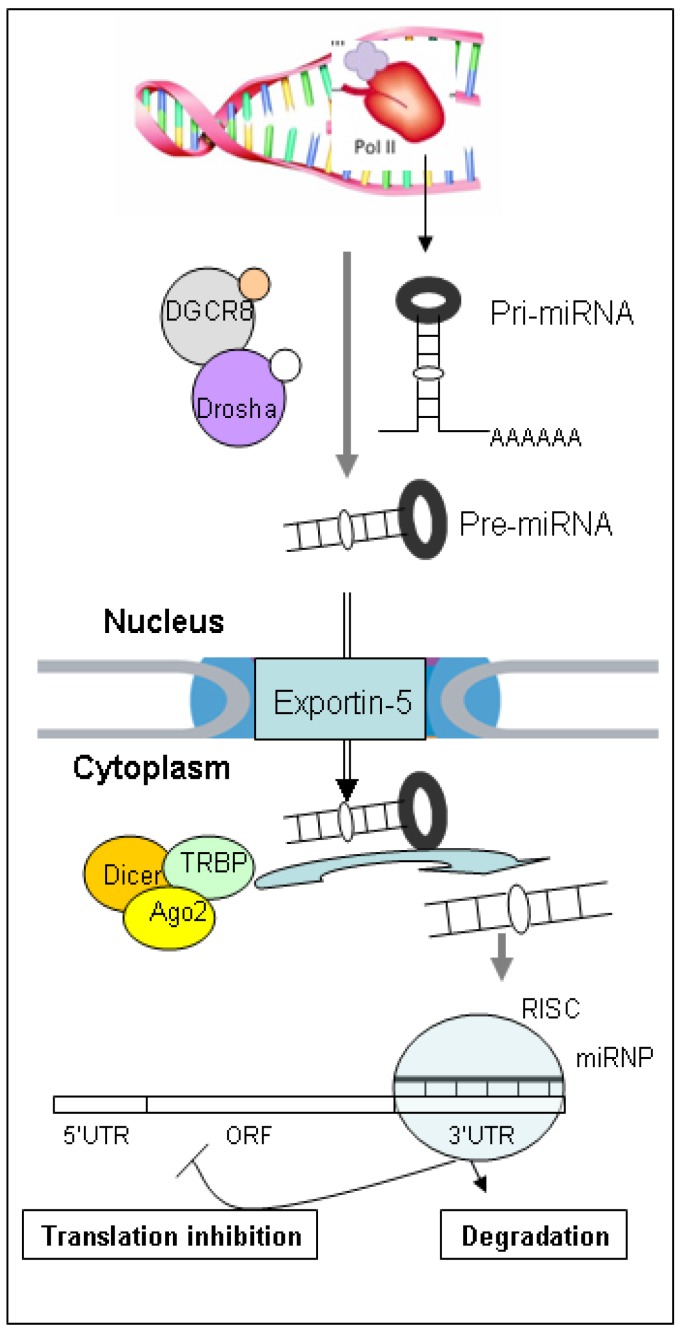
miRNA biosynthesis and function. ORF, open reading frame; RISC, RNA-induced silencing complex; UTR, untranslated region.

miRNAs are a class of conserved small, non-coding RNA molecules measuring 17–25 nucleotides in length. Since their discovery in 1993 [19], miRNAs have been shown to play important roles in regulating gene expression by either repressing the translation of or causing the degradation of multiple-target mRNAs [20,21,22]. miRNA species account for 1%–3% of the mammalian genome [23]. miRNA transcripts are generated from the stem-loop precursors transcribed by RNA polymerase II to form the primary precursor miRNA (pri-miRNA). Pri-miRNAs initially contain a cap structure at the 5' end and a 3' poly(A) tail. In the nucleus, the stem loop is asymmetrically cleaved by a complex comprising the RNase III Drosha and its cofactor DGCR8 to produce the precursor miRNA (pre-miRNA), which is approximately 70 nucleotides in length. The pre-miRNA is transported to the cytoplasm, a process mediated by the nuclear transport receptor exportin-5 and the nuclear protein Ran-GTP, and the exported pre-miRNA is cleaved by Dicer to produce a short duplex molecule. One of the strands of the duplex is selected to form the miRNA-induced silencing complex, which can repress translation or target mRNA cleavage (Figure 1). Argonaute (Ago) proteins have been implicated in the transcriptional and post-transcriptional gene silencing of targeted mRNA [23,24,25]. Since the discovery in *Caenorhabditis elegans* that the *lin-4* and *let-7* RNAs regulate developmental timing [19,26], miRNAs have been known to have critical regulatory functions in development, proliferatin, differentiation, apoptosis and stress response [23,27]. An increasing number of studies have established the regulatory roles of miRNAs in complex genetic networks underlying various cellular pathways, indicating that oncogenic miRNAs might be involved in the genetic networks regulating the functional pathways deregulated in cancer cells [27].

## 3. Tumor Tissue miRNAs

On the basis of tissue-specific deregulation of miRNA expression in cancer, multiple studies have explored the potential usefulness of miRNA expression profiles and have found that miRNAs from tumor tissue could play an important role in predicting the tissue of origin for tumors of unknown origin or in poorly differentiated tumors and to distinguish subtypes of tumors. Combination analysis of Illumina/Solexa deep sequencing and miRNA microarray can not only exhibit high sequence counts and concordant differential expression in prostate cancer [28] and breast cancer [29] but also identify miRNA expression signatures that may serve as accurate tools for the diagnosis and prognosis of cancer including response to therapeutic drugs. Large tissue specimens are not needed for accurate miRNA detection since their expression can be easily measured in biopsy specimens. miRNAs have been measured in formalin-fixed paraffin-embedded tissues of hepatocellular carcinoma [30], lung cancer [31], melanoma [32], renal tumor [33] and papillary thyroid carcinoma [34]. These specimens have also been used reliably for miRNA deep-sequencing analysis in renal cell carcinoma [35] and for locked nucleic acid-based miRNA quantitative polymerase chain reaction (qPCR) analysis of many tumor tissues [36].

miRNA microarray analysis has been performed to compare miRNA levels in bone marrow from four breast cancer patients with recurrent disease and four patients without recurrence. The results revealed that accumulation of *miR-21* and *miR-181a* in bone marrow appears to be associated with prognosis in breast cancer patients and plays a predictive role in determining the incidence of recurrence and metastasis [37]. High expression level of *miR-21* was also associated with poor survival and therapeutic outcome in colon adenocarcinoma patients [38] and correlated with outcome in pancreatic ductal adenocarcinoma patients treated with gemcitabine [39]. In contrast, liver cancer patients whose tumors had low *miR-26* expression had shorter overall survival but a better response to interferon therapy than did patients whose tumors had high expression of this miRNA [40]. Reduced *let-7* expression in human lung cancers was associated with significantly shorter survival after resection [41]. Expression of *miR-126* and *miR-335* was lost in the majority of primary breast tumors from patients who relapse, and the loss of expression of either miRNA was associated with poor distant metastasis-free survival [42]. These results suggest that miRNAs could be useful biomarkers of prognosis and therapy response.

miRNAs can act as either oncogenes or tumor suppressors, depending on the cellular context and the genes targeted in each instance [43,44,45,46]. The mir-17-92 cluster was a potential oncogene in human B-cell lymphomas [44]. The *let-7* family was the first group of oncomirs shown to regulate the expression of the *Ras* gene [46]. *miR-21*, as an oncomir, targets *PTEN* [47,48] and *SPRY2* [49] and promotes invasion and migration, which drives tumorigenesis through inhibition of negative regulators of the *Ras*/*MEK*/*ERK* pathway [50]. Tumor-suppressor miRNAs such as *miR-96* and *miR-34a* are repressed in primary tumors [43,45]. These miRNAs are expressed at relatively higher levels in differentiated tissues and at reduced levels in cancer. *miR-96* decreased pancreatic cancer cell invasion and migration and slowed tumor growth, which was associated with *KRAS* downregulation [45]. *miR-34a* was repressed in primary neuroblastoma tumors and cell lines and targeted *E2F3*, a potent transcriptional inducer of cell-cycle progression [43]. *miR-34a* also reduced choriocarcinoma cell proliferation and invasiveness through its inhibitory effect on DLL1 [51]. Lower expression of these miRNAs possibly reflects loss of differentiation, which is a hallmark of cancer.

## 4. Circulating miRNAs

The assays for cfDNA, miRNAs and caspase activity in blood might be developed as novel minimally invasive diagnostic tools for the detection and risk assessment of lung cancer, provided that their clinical utility can be confirmed in larger prospective trials [52]. In a recent study lung cancer patients were distinguished from healthy individuals by combined analysis of the concentrations of cfDNA, serum miRNAs and caspase activity, with the levels of *miR-10b*, *miR-141* and *miR-155* found significantly higher in patients with malignant disease than those with benign disease [52].

Some studies have analyzed the clinical relevance of circulating miRNAs in peripheral blood for diagnosis and prognosis and demonstrated the potential of serum and plasma circulating nucleic acids to act as novel noninvasive biomarkers for the early diagnosis of various cancers and other diseases [17,53,54,55]. In particular, Mitchell *et al.* clearly showed that circulating miRNAs originate from cancer tissues and are protected from endogenous RNase activity [53], reflecting the potential of circulating miRNAs to serve as circulating biomarker candidates of cancer.

miRNA microarray, miRNA real-time qPCR array (qRT-PCR array), and next-generation sequencing (NGS) technology have been utilized to screen for circulating miRNAs and generated miRNA signatures from body fluids. qRT-PCR is a popular method for gene expression quantification of miRNA [56]. Several companies, including Life Technologies, Qiagen and Exiqon, have developed qRT-PCR arrays to profile a large set of miRNAs simultaneously and for signature-based analyses [57]. Pre-amplification of miRNAs enables qPCR-based miRNA detection to be highly multiplexed and high throughput. miRNA qRT-PCR array analysis is relatively straightforward, with analysis outputs providing either absolute quantification or relative quantification [56]. Normalization of miRNA data is critical to interpreting clinical significance and developing miRNAs as tumor markers. The careful selection of reference RNAs plays a crucial role in miRNA expression studies. To date, the most commonly used references are *U6 snRNA* (*RNU6B*) [58,59,60], *RNU19*, *RNU43*, *RNU47*, *RNU49*, *U1* [61], *RNU44*, *RNU48*, *U75* [62], *5S RNA* [63,64], *18S RNA* [65], *GAPDH* [66] and some reference miRNAs such as *miR-16* [67,68], *let-7a* [67] and *miR-106b* [65] have also been utilized. Although certain miRNAs could be used as universal references, lack of stability of some reference RNAs make them untenable as reference for normalization purpose [69]. These confounding factors for reliable normalization of circulating miRNAs have led to the use of “spiked in” synthetic miRNAs for quantification of data [53]. For example, *RNU44*, stably expressed in endometrioid endometrial cancer tissues, could be used for normalization in miRNA qPCR studies [62], but due to the association with prognosis in head and neck squamous cell carcinoma and breast cancer cannot be used as a reference RNA in these malignancies [70]. *5S* and *RNU6B* are commonly used to normalize miRNA qRT-PCR data [63,64,71]. However, *RNU6B* was associated with clinicopathological factors in relevant cancer samples [70]. Moreover, *miR-191*, *miR-103* and *miR-17-5p* were found superior to *5S* or *RNU6B* as normalizing controls in normal and cancerous human solid tissues [69]. *RNU6B* was less stably expressed than *let-7a* and *miR-16* in breast cancer [69,71]. Expression differences between the sample groups and their dependence on the intactness this RNA are arguments against use of *RNU6B* as the reference RNA in miRNA expression studies in renal cell carcinoma in order to avoid misleading results [61].

Proper normalization of miRNA quantification requires a careful choice and validation of reference in the representative sample of the studied population [62,72]. Microarray-based selection and qRT-PCR-based validation of miRNA reference for normalizing miRNA qRT-PCR data is now a simple and effective approach [61]. The stability of candidate references can be assessed using Normfinder, GeNorm and BestKeeper software programs [60,62,73,74]. NormFinder employs a model-based approach that, in addition to the overall expression level variation, takes into account the intra- and intergroup variation of the candidate normalization reference to evaluate the expression stability. The GeNorm program can calculate the stability values (M-values) of a gene based on the average pairwise variation between all studied transcripts. High gene expression variability results in high M values, which indicates low expression stability of the candidate reference [75]. The number of references required for optimal normalization performed by GeNorm is used to calculate the pairwise variation between sequential normalization factors [62]. BestKeeper determines the stability of candidate reference, which is based on the coefficient of variation multiplied by 100 [62]. On the other hand, Pritchard *et al.* found the effect of hemolysis on the quantification and normalization of circulating miRNAs in body fluid because circulating tumor-associated miRNAs were found highly expressed in blood cells [76] and the presence of hemolysis in plasma samples affected the levels of *miR-16* and *miR-451* in body fluid [77]. Thus, circulating miRNA results must be cautiously interpreted, as they may reflect a blood cell-based phenomenon rather than a cancer-specific origin, and accurate normalization in miRNA expression studies using validated reference remains a formidable challenge.

The microarray platform also enables the simultaneous analysis of all human miRNAs and can be easily redesigned to include newly identified miRNAs. miRNA array allows each sample to be profiled for a large set of miRNAs. The ability to profile thousands of known transcripts is the main advantage of microarrays [78]. Several companies, such as Affymetrix, Agilent Technologies, Illumina, LC Sciences and Exiqon, have miRNA array systems. But both qRT-PCR array and miRNA array analyses are limited in that they profile only known or putative miRNAs, and base sequence data are not always accounted for. NGS has become an increasingly popular method for miRNA profiling [79], which provides quantification of a variety of small RNA (about 10–40 nt) species and accurate quantification and differential expression with a wide-dynamic range [80]. Several next-generation platforms can produce paired-end reads, which are joined together to form longer contiguous reads (known as contigs) by a computer program (known as an assembler) for miRNA sequence analysis [81].

TaqMan low-density arrays were first used to analyze human miRNAs in plasma from non-small cell lung cancer (NSCLC) patients and controls, and selected miRNA signatures such as *let-7f*, *miR-20b*, *miR-30e-3p*, *miR-223* and *miR-301* were validated independently by qRT-PCR in plasma and correlated with pathologic parameters and survival [82]. Not only was a 34-miRNA signature identified in serum of the patients with early stage NSCLC using miRNA qRT-PCR array assays [83], but *miR-126* and *miR-183* was also identified as candidate potential circulating biomarkers for metastatic NSCLC [84]. Solexa NGS followed by individual qRT-PCR assays was used with NSCLC patients, which demonstrated that a set of 11 serum miRNAs was differentially expressed between patients with longer and shorter survival [85]. Following computed tomography screening, a circulating 9-miRNA signature (*miR-221*, *miR-660*, *miR-486-5p*, *miR-28-3p*, *miR-197*, *miR-106a*, *miR-451*, *miR-140-5p*, and *miR-16*) was determined to indicate a risk of aggressive lung cancer, and *miR-486-5p* was found down-regulated in plasma of patients with a poor outcome [86]. A logistic regression model with the best prediction was defined on the basis of four miRNAs (*miRNA-21*, *miR-126*, *miR-210*, and *miR-486-5p*) yielding high sensitivity and specificity in distinguishing NSCLC patients from the healthy control group. This panel of miRNAs exhibited 73.33% sensitivity and 96.55% specificity in identifying stage I NSCLC patients [87]. In a study comparing the levels of circulating miRNAs in plasma samples from patients with early stage breast cancer and matched healthy controls using microarray-based expression profiling and qRT-PCR analysis, miRNAs of 18–31 nt in length were reported to be differentially expressed in cancer patients compared with healthy subjects [88]. Circulating miRNA expression signatures in human serum for five types of human cancer (prostate, colon, ovarian, breast and lung) have been evaluated using a human miRNA high-density microarray, and 15 circulating miRNAs were found to be elevated in serum from prostate cancer patients compared with normal donor serum [89]. Circulating *miR-375* and *miR-141* were differentially quantified in men with metastatic prostate cancer compared with individuals with non-metastatic disease and significantly correlated with adverse risk factors [90,91,92,93]. Five serum miRNAs (*miR-21*, *miR-92*, *miR-93*, *miR-126*, and *miR-29a*) were significantly overexpressed in a set of 19 samples from epithelial ovarian cancer patients before therapy compared with 11 healthy controls [94]. Elevated levels of circulating *miR-200* family members correlated with serous ovarian cancer [95], while *miR-132*, *miR-26a*, *let-7b*, and *miR-145* miRNAs were significantly repressed in the serum of serous ovarian cancer patients [96]. Solexa NGS analysis revealed that 19 circulating miRNAs were markedly elevated in the serum of gastric cancer patients and that the expression level of five serum miRNAs (*miR-1*, *miR-20a*, *miR-27a*, *miR-34* and *miR-423-5p*) was correlated with gastric cancer tumor stage [97]. Among patients with gastric cancer, those who had lymph node metastasis had higher serum levels of *miR-21*, *miR-146a* and *miR-148a* compared with patients without lymph node metastasis, which implied that these miRNAs might be candidates for noninvasive biomarkers to predict lymph node metastasis in patients with gastric cancer [98]. NGS analysis demonstrated that 25 were up-regulated in esophageal squamous cell carcinoma patients compared with their matched controls and qRT-PCR analysis further identified a profile of seven serum miRNAs (*miR-10a*, *miR-22*, *miR-100*, *miR-148b*, *miR-223*, *miR-133a*, and *miR-127-3p*) as esophageal squamous cell carcinoma biomarkers [99]. Another seven-miRNA panel (*miR-122*, *miR-192*, *miR-21*, *miR-223*, *miR-26a*, *miR-27a* and *miR-801*) could differentiate between patients with hepatocellular carcinoma (HCC) from healthy patients [100]. Recently, four miRNAs (*miR-150*, *miR-30c*, *miR-483-5p* and *miR-520b*) detectable in all samples with varying expression levels were validated, and the combination of plasma *miR-483-5p* level and hepatitis C virus status were determined to be a signature distinguishing HCC cases from controls [101]. Circulating *miR-21*, *miR-155* and *miR-210* have been found in serum or plasma from patients with different cancers. The combined expression analysis of *miR-21*, *miR-210*, *miR-155* and *miR-196a* in plasma can discriminate pancreatic adenocarcinoma patients from controls [102]. Elevated circulating *miR-21* has been found in the plasma or serum of patients with breast cancer [103,104,105], NSCLC [106], pancreatic ductal adenocarcinoma [102], gastric [98,107], lung [87,108], and ovarian cancer [94,109,110], HCC [100,111], prostate cancer [112,113], esophageal squamous cell carcinoma [114], and DLBCL [17] and identified as an independent prognostic factor for breast and lung cancer [103,106]. Elevated circulating *miR-21* was also associated with relapse-free survival in DLBCL patients [17] and shown to be involved in docetaxel-resistance in hormone-refractory prostate cancer [53]. Circulating *miR-155* has been detected in blood of patients with DLBCL [17], breast cancer [54,105,115,116] and lung cancer [117]; urine of patients with bladder cancer [118]; and pancreatic juice of PDAC patients [119]. *miR-210*, highly expressed in serum or plasma of patients with lung cancer [87,120], clear cell renal cell carcinoma [121], DLBCL [17] and pancreatic cancer, was confirmed to be a novel hypoxia marker [122]. On the other hand, the levels of plasma *miR-601* and *miR-760* are significantly decreased in patients with colorectal neoplasia (carcinomas and advanced adenomas) compared with healthy controls [123]. Intriguingly, some circulating miRNAs have revealed discordant expression patterns in plasma or serum from specific cancer types. For example, the level of *miR-92a* was significantly higher in plasma or serum samples from patients with advanced-stage colorectal cancer [124,125], breast cancer [104] and ovarian cancer [94] but was down-regulated in plasma from acute leukemia [126], bladder cancer [127] or HCC patients compared with healthy controls [128].

Interestingly, highly expressed circulating miRNAs from cancer patients have been reported to return to a normal level after tumor resection. For example, serum levels of up-regulated miRNAs such as *miR-21* and *miR-106b* were significantly higher in pre-operative plasma from patients with gastric cancer than before resection [98]. High levels of *miR-500* were found in the serum of patients with HCC, yet the circulating *miR-500* returned to normal after tumor resection in three of the patients [129]. Increased systemic *miR-195* levels in breast cancer patients were observed in breast tumors, and circulating levels of *miR-195* and *let-7a* were also decreased after resection of the tumor [130]. Surgical removal of the primary tumor also coincided with reduction in plasma *miR-184* levels in patients with squamous cell carcinoma of the tongue [131]. The expression levels of *miR-96* and *miR-183* were significantly lower in urine collected after surgery in urothelial carcinoma patients [132]. These findings have suggested that the level of circulating miRNAs reflect the expression level of tumor miRNAs.

Although the majority of studies have assessed circulating miRNAs in serum and plasma, recent studies have confirmed the potential use of tumor-specific miRNAs as diagnostic markers for cancer in other body fluids, such as urine [118,132,133,134,135,136,137], saliva [138], pancreatic juice [119] and cyst fluid [139] (Table 1). The presence of miRNAs in body fluids may represent a gold mine of noninvasive biomarkers in cancer. For instance, the significant increases in *miR-96* and *miR-183* expression in urine were associated with advanced tumor grade and pathological stage in urothelial carcinoma patients [132]. Urine levels of *miR-1236*, *miR-374a* and *miR-767-3p* were increased in cancer patients in general, whereas *miR-200a* and *miR-891b* were not observed in samples from patients with bladder urothelial cancers [133]. *miR-125a* and *miR-200a* were found to be present in significantly lower levels in the saliva of patients with oral squamous cell carcinoma compared with matched healthy controls [138]. miRNAs have also been detected in tears, breast milk, bronchial lavage, colostrum, seminal, amniotic, pleural, peritoneal, and cerebrospinal fluids [18,133]. Unsupervised hierarchical clustering analysis of commonly expressed miRNAs reveals that the miRNA spectrum in plasma is different from that of most other body fluids [133]. These findings might be useful if a correlation between specific miRNA levels in body fluids and various disease states is proven.

**Table 1:** Circulating tumor-associated miRNAs as potential biomarker for cancers.

Cancer	Study Design	Body Fluid	Differentially Expressed miRNA	Method	Ref.
Lung cancer	Tumor (NSCLC) *vs.* normal	Serum	*miR-25*, *miR-223*	NGS, qRT-PCR	[55]
	Tumor (NSCLC) *vs.* normal, prognosis of NSCLC	Plasma	*miR-30e-3p*, *let-7f*	qRT-PCR array, qRT-PCR	[82]
	Symptomatic AC, SCC *vs.* benign lung disease	Serum	34-miRNA signature	qRT-PCR array	[83]
	Stage I/II *vs.* IV NSCLC	Serum	*miR-126*, *miR-183*	qRT-PCR	[84]
	Prognosis of NSCLC	Serum	*miR-486*, *miR-30d*, *miR-1*, *miR-499*	NGS, qRT-PCR	[85]
	Tumor (NSCLC) *vs.* normal, prognosis of NSCLC	Plasma	9-miRNA signature	qRT-PCR array	[86]
	Tumor (NSCLC) *vs.* normal, stage I NSCLC	Plasma	*miRNA-21*, *miR-126*, *miR-210*, *miR-486-5p*	qRT-PCR	[87]
	Tumor (NSCLC) *vs.* normal, tumor-node metastasis stage	Serum	*miR-21*	qRT-PCR	[106]
	Tumor (NSCLC) *vs.* normal, Prognosis of NSCLC	Serum	*miR-21*, *miR-141*, *miR-200c*	qRT-PCR	[108]
	Adenocarcinoma *vs.* normal	Plasma	*miR-21*, *miR-155*	miR array	[117]
Breast cancer	Tumor *vs.* healthy controls	Serum	*miR-155*	qRT-PCR	[54]
	Tumor *vs.* healthy controls	Plasma	*let-7c*, let-7d*, *miR-589*, miR-425*	miR array, qRT-PCR	[88]
	Different stages of tumor *vs.* healthy controls	Serum	*miR-21*	qRT-PCR	[103]
	Tumor *vs.* healthy controls	Serum	*miR-29a*, *miR-21*	qRT-PCR	[104]
	Tumor *vs.* healthy controls	Serum	*miR-21*, *miR-106a*, *miR-155*, *miR-126*, *miR-199a*, *miR-335*	qRT-PCR	[105]
	Primary breast cancer, metastatic disease *vs.* healthy women	Serum	*miR-10b*, *miR-155*, *miR-34a*	qRT-PCR	[115]
	Primary breast cancer, metastatic disease *vs.* healthy women	Serum	*miR-34a*, *miR-93*, *miR-155,miR-373*	qRT-PCR	[116]
	Tumor *vs.* healthy controls, tumor resection	Blood	*miR-195*, *let-7a*	qRT-PCR	[130]
	Prognostic prediction of PCa	Serum	12-miRNA signature	NGS, miR array, qRT-PCR	[28]
	Metastatic PCa *vs.* benign	Plasma	*miR-100*, *miR-125b*, *miR-141*	qRT-PCR	[53]
	All stage 3 and 4 PCa *vs.* healthy controls	Serum	15-miRNA signature	miR array	[89]
	Metastatic PCa *vs.* benign	Serum	*miR-141*, *miR-375*	qRT-PCR	[90]
Prostate cancer	Metastatic *vs.* localized PCa	Plasma	*miR-141*, *miR-375*, *miR-181a-2*	qRT-PCR	[91]
	Metastatic *vs.* localized PCa	Serum	miR-9*, *miR-141*, *miR-200b*, *miR-375*, *miR-516a*	qRT-PCR	[92]
	Metastatic *vs.* localized PCa	Serum	*miR-375*, miR-378*, *miR-141*	miR array, qRT-PCR, miR array	[93]
	Metastatic PCa *vs.* benign	Serum	*miR-21*	qRT-PCR	[112]
	Prognostic prediction of PCa	Plasma	*miR-20a*, *miR-21*, *miR-145*, *miR-221*	qRT-PCR	[113]
Ovarian cancer	Epithelial OC *vs.* healthy controls	Serum	*miR-21*, *miR-29a*, *miR-126*, *miR-92*, *miR-93*	miR array, qRT-PCR	[94]
	High-grade serous OC *vs*. normal	Serum	*miR-200* family	qRT-PCR	[95]
	Serous OC *vs.* normal	Serum	*miR-132*, *miR-26a*, *let-7b*, *miR-145*	miR array, qRT-PCR	[96]
	Various stages of OC *vs.* benign disease	Serum	8-miRNA signature, *miR-21*, *miR-141*, *miR-200*	miR array	[109]
	Endometriosis, and endometriosis-associated OC *vs.* normal	Plasma	10-miRNA signature	miR array, qRT-PCR	[110]
Bladder cancer	Bladder cancer *vs.* healthy controls	Urine	*miR-200* family, *miR-155*, *miR-192*, *miR-205*	qRT-PCR	[118]
	MIBC and non-MIBC *vs.* healthy controls	Plasma	*miR-92*, *miR-33*	qRT-PCR	[127]
	UC *vs.* healthy controls, tumor grade and stage	Urine	*miR-96*, *miR-183*	qRT-PCR	[132]
	Tumor *vs.* healthy controls	Urine	*miR-1236*, *miR-374a*, *miR-767-3p*, *miR-200a*, *miR-891b*	qRT-PCR array	[133]
	UC *vs.* healthy controls	Urine	*miR-135b*, *miR-15b*, *miR-1224-3p*	qRT-PCR	[134]
	Low-grade, high-grade BCa *vs.* healthy controls	Urine	*miR-126*, *miR-152*	qRT-PCR	[135]
	Invasive tumors *vs.* healthy controls	Blood/urine	*miR-26b-5p*, *miR-144-5p*, *miR-374-5p*, *miR-618*, *miR-1255b-5p*	miR array, qRT-PCR	[136]
Pancreatic cancer	PDAC *vs.* healthy controls	Plasma	*miR-21*, *miR-210*, *miR-155*, *miR-196a*	qRT-PCR	[102]
	PaCa *vs.* chronic pancreatitis	Pancreatic juice	*miR-21*, *miR-155*	qRT-PCR	[119]
	PaCa *vs.* healthy controls	Plasma	*miR-210*	qRT-PCR	[122]
	High-grade IPMN *vs.* low-grade IPMN	Cyst fluid	18-miRNA signature	qRT-PCR array, qRT-PCR	[139]
Gastric cancer	GC *vs.* healthy controls	Serum	*miR-1*, *miR-20a*, *miR-27*a, *miR-34*, *miR-423-5p*	NGS, qRT-PCR	[97]
	GC stages; LN metastasis *vs.* LN negative	Serum	*miR-21*, *miR-27a*, *miR-106b*, *miR-146a*, *miR-148a*, *miR-223*	qRT-PCR	[98]
	GC *vs.* healthy controls	Plasma	*miR-106a*, *miR-106b*, *miR-21*, *let-7a*, *miR-17-5p*	qRT-PCR	[107]
Liver cancer	HCC, chronic hepatitis B *vs.* healthy controls	Plasma	*miR-122*, *miR-192*, *miR-21*, *miR-223*, *miR-26a*, *miR-27a*, *miR-801*	miR array, qRT-PCR	[100]
	HCC *vs.* healthy controls	Plasma	*miR-150*, *miR-30c*, *miR-483-5p*, *miR-520b*	qRT-PCR array, qRT-PCR	[101]
	HCC, chronic hepatitis *vs.* healthy controls	Serum	*miR-21*, *miR-122*, *miR-223*	qRT-PCR	[111]
	HCC *vs.* healthy controls	Plasma	*miR-92a*	qRT-PCR	[128]
	HCC *vs.* healthy controls	Serum	*miR-500*	qRT-PCR	[129]
Colorectal cancer	CRC, advanced adenoma *vs.* healthy controls	Plasma	*miR-601*, *miR-760*	qRT-PCR array, qRT-PCR	[123]
	CRC, GC, IBD *vs.* healthy controls	Plasma	*miR-92*, *miR-17-3p*, *miR-135b*, *miR-222*, *miR-95*	qRT-PCR array, qRT-PCR	[124]
	CRC *vs.* healthy controls	Plasma	*miR-29a*, *miR-92a*	qRT-PCR	[125]
Oral cancer	SCC *vs.* healthy controls	Plasma	*miR-184*	qRT-PCR	[131]
	SCC *vs.* healthy controls	Saliva	*miR-125a*, *miR-200a*	qRT-PCR array, qRT-PCR	[138]
Esophageal cancer	Stage I/II ESCC patients *vs.* healthy controls	Serum	*miR-10a*, *miR-133a*, *miR-22*, *miR-100*, *miR-1248b*, *miR-127-3p*, *miR-223*	NGS, qRT-PCR	[99]
	ESCC *vs.* healthy controls	Plasma	*miR-21*, *miR-375*	qRT-PCR	[114]

NGS, next-generation Solexa sequencing; qRT-PCR, quantitative real-time polymerase chain reaction; miR, microRNA; NSCLC, non-small cell lung cancer; AC, adenocarcinoma; SCC, squamous cell carcinoma; PCa, prostate cancer; OC, ovarian cancer; UC, urothelial carcinoma; MIBC, muscle-invasive bladder cancer; PDAC, pancreatic ductal adenocarcinoma; PaCa, pancreatic cancer; IPMN, intraductal papillary mucinous neoplasm; GC, gastric cancer; LN, lymph node; HCC, hepatocellular carcinoma; CRC, colorectal cancer; IBD, inflammatory bowel disease; SCC, squamous cell carcinoma; ESCC, esophageal squamous cell carcinoma.

## 5. Extracellular Circulating miRNAs

Although the level of circulating miRNAs may well reflect the expression level of tumor miRNAs, the release mechanism of circulating miRNAs as an active process is largely unclear. Extracellular RNA is most likely protected within protein or lipid vesicles, or possibly apoptotic bodies. Circulating miRNAs in body fluids and extracellular fluid compartments have hormone-like effects, which lead to widespread consequences [18]. miRNAs also can be passively leaked from apoptotic or necrotic cells, which has been shown to occur after heart tissue injury [140,141]. The secretion of miRNAs by cells is associated with the microenvironment of cells. It has been shown that miRNA can bind to specific proteins and associates with multivesicular bodies (MVBs) and exosomes. Exosomes are small membrane vesicles of endocytic origin and can be formed through inward budding of endosomal membranes, giving rise to intracellular MVBs that later fuse with the plasma membrane. After fusion, MVBs can release exosomes into body fluids [18,142]. Since exosomes are secreted by most cells in culture [142], theoretically all species of miRNAs could be detected in circulation. The wide range of sources of circulating miRNAs makes it possible for circulating miRNAs to reflect every aspect of human physiological status and, therefore, provides an advantage of being better biomarkers than other circulating molecules, such as DNA and RNA [143].

Extracellular miRNAs have recently been detected in exosomes or microvesicles isolated from peripheral blood and culture media of several cell lines [109,144,145,146], which identified that normal non-tumor cells also physiologically produce miRNA- or mRNA-containing exosomes or microvesicles. Several methods for isolating circulating miRNAs from microvesicles in human plasma have been developed, such as ultracentrifugation [145], ExoQuick precipitation [146] and immunoprecipitation of miRNAs with RNA-binding protein [147]. It is important to note that the levels of circulating miRNAs *miR-92a* and *miR-486-5p* were found to be significantly influenced by the exosome isolation method [148], indicating that the exosomal miRNA profiles are affected by the different extracellular vesicle isolation methods. ExoQuick precipitation, using a proprietary resin developed by Systems Biosciences (Mountain View, CA, USA), isolates and purifies all microvesicles from body fluids and has been shown to result in much higher recovery and purity of microvesicles from ascites when compared to the other isolation methods [146,149].

Extracellular circulating miRNAs may occasionally be derived from normal or tumor-lysed cells in body fluids as passive release mechanism [55,150]. Circulating miRNAs can also be packed into microparticles and exosomes and released by tumor cells or circulating microvesicles via shedding of microvesicles as active secretion mechanism [151]. Exosomes containing mRNA, miRNAs, and angiogenic proteins released by tumor cells has been demonstrated [152]. With the capacity to efficiently transfer between cells, circulating miRNAs, particularly cell-derived microvesicle-contained miRNAs, may be an essential part of cellular responses to exogenous challenges [143,153]. Fabbri *et al.* found that circulating tumor-associated *miR-21* and *miR-29a* could induce a Toll-like receptor (TLR)-mediated prometastatic inflammatory response by binding to TLR receptors playing key regulator role for the tumor microenvironment [154].

The advantage of extracellular circulating miRNAs representing a whole organism’s state might in fact become a disadvantage as biomarkers need to distinguish only between a healthy state and one disease state, and not be confounded with a different disease. In order to use miRNAs as biomarkers in cancer, it is important that the source of the tumor-specific miRNAs in body fluids be determined and that a signature capable of differentiating between disease and healthy states be established. Also, it is necessary to clarify whether the differential expression between tumor and normal tissues is related solely to the tumor or is a response mediated by the affected organ or system. Thus, we need to clarify the special signature of tumor-associated extracellular circulating miRNAs in different tumors, circulating miRNAs may be derived from circulating tumor cells (CTCs), primary or metastatic tumor cells as well as nonmalignant cells such as platelets, or damaged nonmalignant cells entering the circulation.

## 6. CTC-Associated miRNAs

Identification of tumor cells within the blood stream, which are referred to as circulating tumor cells (CTCs), have lately been considered a valuable resource for developing reliable surrogate biomarkers. CTCs, which have been found in the blood of cancer patients [155], were first thought to be viable metastatic precursors capable of initiating a clonal metastatic lesion. Though extraordinarily rare, CTCs have been detected in a majority of epithelial cancers, including breast, lung, liver, prostate, kidney, pancreatic, esophageal and colorectal cancers [156,157,158]. Translational research has implicated CTCs in several biological processes, including epithelial-to-mesenchymal transition [159], and so these cells are being integrated into clinical trial designs as a surrogate for genotypic and phenotypic markers to correlate with the outcome of molecularly targeted therapies. Efforts are being made to develop reliable procedures for the sensitive and specific detection of CTCs, either at the protein level utilizing antibody-based cell staining methods or at the mRNA level by employing qRT-PCR techniques. Utilizing whole-genome oligonucleotide microarrays and a TaqMan low-density array, researchers recently developed a panel of six candidate gene markers for the detection of CTCs in the blood of breast cancer patients; these markers might also serve as potential markers of selected CTCs derived from endometrial, cervical and ovarian cancers [158]. The isolation and subsequent characterization of CTCs provide the opportunity to bypass the problems associated with obtaining metastatic tissue. CTCs have already been characterized for gene amplification and the expression levels of specific proteins, mRNAs and miRNAs [153].

Most studies have measured miRNAs in the serum, plasma or exosome fraction of blood rather than in whole blood. Using serum or plasma does, for the most part, eliminate the leukocyte background present in whole blood, but most miRNAs measured in these fractions may not actually be derived from circulating epithelial cells [53] and cellular miRNA expression patterns can differ from miRNA patterns released into the blood [55]. These studies have raised the concern that circulating cell-free miRNAs may not reliably reflect the miRNA profile of metastatic or primary tumor tissue, indicating that measuring CTC-associated miRNAs would be preferable. Efforts are being made to develop a CTC isolation method that provides a purer CTC fraction for downstream analysis. Obtaining a higher purity of the enriched CTC fraction through more specific CTC isolation techniques would eliminate the need to only measure epithelium-specific genes and miRNAs [153]. The development of enrichment methods that provide a purer CTC fraction would likely simplify the measurement of CTC-associated miRNAs. Due to the low numbers of CTCs in circulation [160], more sensitive RNA isolation and unbiased pre-amplification techniques may be needed.

## 7. Relationship between Circulating miRNAs and Tumor Tissue miRNAs

Several studies have assessed the relationship between serum or plasma miRNAs and tissue miRNAs [92,105,109,161]. Identifying the correlation between circulating miRNAs and tissue miRNAs would support the hypothesis that circulating miRNAs can serve as ideal biomarkers for various diseases. Circulating miRNAs are actively released by cells, should reflect the miRNome of the tumor tissues, many miRNAs show the same trend of alteration (an increase or a decrease) in the plasma or serum and tumor tissues of patients in various types of cancer. In a study of whether plasma miRNAs could be released from primary gastric tumors, a comparison between the expression of miRNA in plasma and primary gastric cancer tumor tissues revealed similar trends in the expression of miRNAs in almost all cases, indicating that the level of plasma miRNAs might reflect the expression level of tumor miRNAs [107]. In that study, the circulating concentrations of *miR-21* and *miR-106b* were significantly reduced post-operatively in patients with high pre-operative plasma *miR-21* and *miR-106b*. Another study demonstrated that plasma samples and the corresponding primary NSCLC tissues showed similar trends in the expression of *miR-21*, *miR-126*, *miR-182*, *miR-210*, and *miR*-*486-5p* [120]. The level of *miR-155* was also found to be elevated in plasma of patients with lymphoma [17] and human B cell lymphomas [162]. Taken together, these results indicated significant concordance of miRNA expression levels in plasma and the corresponding tumor tissues.

However, certain tumor suppressor miRNAs show an inverse relationship also between circulating miRNAs and tissue or cell miRNAs, such as *miR-122*, *miR-34a* and *miR-29*. Although the liver-specific *miR-122* is frequently suppressed in primary HCC [163,164], serum *miR-122* is significantly higher in patients with HCC than in healthy controls [110]. Due to aberrant CpG methylation of its promoter, *miR-34a* is commonly silenced in human cancers, such as pancreatic cancer [165], neuroblastoma [43], breast, lung, colon, kidney, or bladder cancer, and primary melanoma [166], but serum *miR-34a* has been reported to be markedly up-regulated in gastric cancer patients [97] and breast cancer patients [116]. *miR-29a* reportedly acts as a tumor suppressor in lung cancer [167], HCC [168], B-cell chronic lymphocytic leukemia [169], breast cancer [170] and ovarian carcinoma [171]. Over-expression of *miR-29a in vitro* decreased the viability of ovarian cancer and breast cancer cells [171]. Mantle cell lymphoma patients with a significantly down-regulated *miR-29* level had a shorter survival time compared with patients with relatively high levels of *miR-29* [172]. On the other hand, *miR-29a* was found significantly elevated in the plasma or serum of patients with advanced colorectal neoplasia [125], breast cancer [104] or ovarian cancer [94]. These results indicate that the inverse relationship between tissue and circulating miRNAs, may reflect an yet unknown biological phenomenon of physiological significance.

## 8. Circulating and Tissue miRNAs as Potential Biomarkers for Cancer

Ideal biomarkers of tumors should be specific, sensitive, and proportional to tumor load. Early studies clearly demonstrated that circulating miRNAs and tissue miRNAs satisfy these criteria. Favorably, the effectiveness of miRNAs as biomarkers for tracing the tissue of origin of cancers of unknown primary origin was demonstrated in 400 paraffin-embedded and fresh-frozen samples from 22 different tumor tissues and metastases [173]. Since the discovery of circulating miRNAs in serum and plasma and the correlation between the expression profile of circulating miRNAs and tumor tissue miRNA, tremendous efforts have been devoted to identify novel circulating miRNA-based noninvasive biomarkers for early tumor detection, diagnosis, and prognosis.

Despite promising developments in the field, circulating and tissue miRNAs as biomarkers for cancer need to be extensively investigated to validate their great potential. First, a simple standard assay for quantifying circulating miRNAs in various types of body fluid should be established. The specificity and sensitivity of circulating and tissue miRNA profile-based biomarkers in a large number of samples should be estimated. Second, specific tumor-associated circulating miRNA signatures will have to be developed as early biomarkers of cancer. The functional roles of these tumor-associated circulating miRNAs should be uncovered. Finally, as methods of circulating miRNA detection and analysis are improved, the assay tools have to be reflected for both technical and analytic aspects. The wide applicability and potential importance of specific miRNAs should help develop reproducible and reliable biomarkers of detection, diagnosis and prognosis for various types of cancer.
